# CAR exosomes derived from effector CAR-T cells have potent antitumour effects and low toxicity

**DOI:** 10.1038/s41467-019-12321-3

**Published:** 2019-09-25

**Authors:** Wenyan Fu, Changhai Lei, Shuowu Liu, Yingshu Cui, Chuqi Wang, Kewen Qian, Tian Li, Yafeng Shen, Xiaoyan Fan, Fangxing Lin, Min Ding, Mingzhu Pan, Xuting Ye, Yongji Yang, Shi Hu

**Affiliations:** 1 Department of Assisted Reproduction, Shanghai Ninth People’s Hospital, Shanghai Jiao Tong University School of Medicine Shanghai, 200011 Shanghai, China; 2 Department of Biophysics, College of Basic Medical Sciences, Second Military Medical University, 200433 Shanghai, China; 3 Team SMMU-China of International Genetically Engineered Machine (iGEM) Competitions, Department of Biophysics, Second Military Medical University, 200433 Shanghai, China; 4 Pharchoice Therapeutics Inc., 201406 Shanghai, China

**Keywords:** Nanoparticles, Targeted therapies

## Abstract

Genetically engineered T cells expressing a chimeric antigen receptor (CAR) are rapidly emerging a promising new treatment for haematological and non-haematological malignancies. CAR-T therapy can induce rapid and durable clinical responses but is associated with unique acute toxicities. Moreover, CAR-T cells are vulnerable to immunosuppressive mechanisms. Here, we report that CAR-T cells release extracellular vesicles, mostly in the form of exosomes that carry CAR on their surface. The CAR-containing exosomes express a high level of cytotoxic molecules and inhibit tumour growth. Compared with CAR-T cells, CAR exosomes do not express Programmed cell Death protein 1 (PD1), and their antitumour effect cannot be weakened by recombinant PD-L1 treatment. In a preclinical in vivo model of cytokine release syndrome, the administration of CAR exosomes is relatively safe compared with CAR-T therapy. This study supports the use of exosomes as biomimetic nanovesicles that may be useful in future therapeutic approaches against tumours.

## Abstract (web summary)

Genetically engineered T cells expressing a chimeric antigen receptor (CAR-T cells) are a promising new treatment for cancer, but are associated with unique toxicities. Here, the authors test CAR-T-cell-derived exosomes as a surrogate for CAR-T cells and show that they can elicit a potent antitumour immune response in preclinical models of breast cancer with reduced signs of cytokine release syndrome compared with CAR-T therapy.

## Introduction

The utilization of genetically engineered autologous or allogeneic T cells expressing chimeric antigen receptors (CARs) or T-cell receptors (TCRs) as cellular immunotherapy is emerging as a promising new treatment method for a broad range of cancers^1,2^, based on the cytotoxic specificity of the T cells towards cancer cells. Typically, CARs consist of a target binding domain, which is an extracellular domain that is specifically expressed by CAR-T cells, a transmembrane domain, and a signalling domain, which is an intracellular domain that provides an activation signal to T cells. The targeting specificity of CARs is usually achieved by antigen-recognition regions in the form of a single-chain variable fragment (scFv) or a binding receptor/ligand in the extracellular domains, while the T-cell-activating function is achieved by the intracellular domain, including a region of the TCR CD3ζ chain that provides ‘signal 1’ and one or more domains from co-stimulatory receptors, such as CD28, OX40 (CD134), and/or 4-1BB (CD137), to provide ‘signal 2’. In current clinical development, targeting CD19^+^ B cell malignancies, which include acute and chronic B-cell leukaemia and B-cell non-Hodgkin lymphomas (NHLs), with anti-CD19 CAR-T cells is one of the most advanced adoptive T-cell therapies and has been approved by the FDA. Data from numerous phase I/II clinical trials conducted at single institutions have indicated that this approach is typically associated with an overall response rate of 50–90% in patients with B-cell malignancies refractory to standard therapies^3,4^.

Despite their efficiency, adoptive T-cell therapies show unique toxicities, which are distinct from those seen with conventional chemotherapies, monoclonal antibodies (mAbs), and small-molecule-targeted therapies. The recognition of toxicity is of utmost importance as the use of these therapies increases. The two most commonly noticed toxic effects in CAR-T immunotherapy are cytokine release syndrome (CRS), which is characterized by high fever, hypotension, hypoxia, and/or multiorgan toxicity, and CAR-T-related encephalopathy syndrome (CRES), which is typically characterized by a toxic encephalopathic state with symptoms of delirium, confusion and, occasionally, cerebral oedema and seizures^5–7^. Rare cases of fulminant haemophagocytic lymphohistiocytosis (HLH) (or macrophage-activation syndrome (MAS)), typically characterized by severe immune activation, lymphohistiocytic tissue infiltration, and immune-mediated multiorgan failure, have also been reported^7–10^. Other redirected T-cell therapies, such as TCR gene therapies and bispecific T-cell-engaging antibodies (BiTEs), as well as preclinical CAR natural killer (NK) cells, have also been reported to induce such toxicity^11–13^. Although a strong response was observed for CAR-T cells in patients with treatment-refractory haematologic malignancies, only modest outcomes have been reported in solid tumours. This result is probably due to a host of obstacles that are encountered in the tumour microenvironment (TME) of solid tumours^14–16^, including intrinsic inhibitory pathways mediated by upregulated inhibitory receptors (IRs) responding to their cognate ligands within the tumour, such as in PD1 signalling^17^.

Exosomes belong to a sub-group of extracellular vesicles (EVs), which are secreted by most cells in the body. EVs can be divided into three sub-groups based on their biogenesis, including exosomes (30–150 nm in diameter), microvesicles (150–1000 nm) and apoptotic bodies (50–2000 nm). Recently, cumulative evidence has emerged, suggesting that membrane vesicles may play a crucial role as mediators of intercellular communication. Exosomes have received the most attention among these vesicles and have also been substantially characterized. Moreover, regarding human T-cell-derived exosomes, their essential role in cytotoxic T lymphocyte (CTL)-target cell interactions was verified in previous studies^18–20^. CTL-derived exosomes contain CTL surface membrane molecules (CD3, CD8 and the TCRs), guaranteeing the unidirectional delivery of the lethal hit to targeted tumour cells. Target cell death resulted from the conjugate formation of interactions between the TCR and proper antigen/MHC combination^18^. The target cell killing effect is induced by lethal chemical compounds in the exosomes, including granzymes, lysosomal enzymes and perforin^19^. TCR activation boosts the production of CTL-derived exosomes, and the presence of the TCR/CD3ζ complex was also demonstrated in the membranes of human-CTL-derived exosomes in another relevant study^21^.

Based on the biological properties of exosomes, exosomes derived from CAR-T cells may exhibit excellent potential for use as direct attackers in immunotherapy. Because exosomes bear functional and structural resemblance to synthetic drug carriers similar to liposomes, exosomes can be further used for drug delivery^22–25^. However, because the targeting specificity of CAR-T cells is determined by an antibody-derived scFv in the CAR structure, exosomes that are isolated directly from the medium of CAR-T cells may be heterogeneous and may lose targeting specificity. These data indicate that the purified CAR-containing exosomes derived from CAR-T cells can be used as cancer-targeting agents and may improve therapeutic efficacy.

Here, we show that exosomes released by CAR-T cells carry CAR on their surface. The CAR-containing exosomes express a high level of cytotoxic molecules and be used as tumour attackers. Assays of in vitro and preclinical in vivo model showed that CAR exosomes do not express PD1, and their antitumour effect cannot be weakened by recombinant PD-L1 treatment. Moreover, the administration of CAR exosomes is relatively safe compared with CAR-T therapy in CRS models. Thus, our data support the use of exosomes as biomimetic nanovesicles that may be an effective strategy for treatment of cancer.

## Results

### Characterization of CAR exosomes

**Fig. 1:**
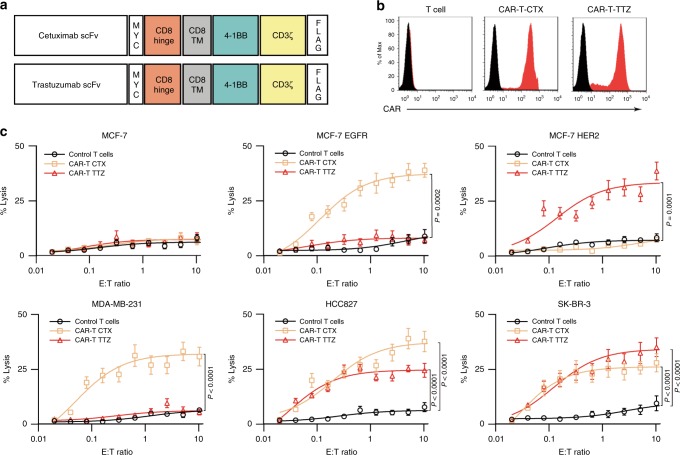
Generation and characterization of CAR-T cells. **a** Vector maps of tested CAR designs. **b** Membrane-bound CAR expression. Forty-eight hours after retroviral transduction, the expression of CAR on human T cells was detected by staining with anti-MYC antibody, followed by flow cytometry analysis. T cells without transduction were used as a negative control. The histograms shown in black correspond to the isotype controls, whereas the red histograms indicate the positive fluorescence. **c** Killing activity of CAR-T cells in response to tumour cells. The cytotoxic activity of CAR-T and control T cells against cancer cell lines was assessed by a ^51^Cr-release assay at the indicated effector-to-target (E:T) ratios. Results shown represent three (**b**) independent experiments. Data are means ± s.d. of five (**c**) independent biological replicates. *P* values are from a two-way ANOVA followed by the Bonferroni post-test (**c**). Source data (**c**) are provided as a Source Data file

Chimeric receptors are designed to contain scFv derived from antibodies that recognize human EGFR and HER2. Cetuximab and trastuzumab were chosen because these antibodies have been found to be safe in patients when administered as targeted drugs. The second-generation CAR design, which includes scFv fused to a CD8a hinge and transmembrane domain and the intracellular domains of human 4-1BB and CD3ζ (or z), was used in our study (Fig. 1a). Next, we used lentiviral vector technology to express the fusion constructs in primary human T cells using clinically validated techniques^26^. The cDNA sequences containing the various fusion constructs were cloned into a third-generation lentiviral vector in which the CMV promoter was replaced with the EF-1α promoter^27^. The receptors were cloned using an extracellular MYC epitope and a C-terminal FLAG tag to permit detection by immunoblotting and flow cytometry. Lentiviral vector supernatants transduced primary T cells with high efficiency (Fig. 1b and Supplementary Fig. 1).

Next, we investigated the antitumour potential of the transduced T cells by standard ^51^Cr-release assays using MCF-7 cells (EGFR- and HER2-negative cells), MCF-7 EGFR cells (a derivative engineered to express EGFR), MCF-7 HER2 cells (a derivative engineered to express HER2), MDA-MB-231 cells, HCC827 cells, and SK-BR-3 cells. The EGFR and HER2 expression levels of these cell lines were measured, and the results are shown in Supplementary Table 1. CAR-T cells transduced with cetuximab scFv (termed CAR-T-CTX) efficiently lysed EGFR-positive cells, such as MCF-7 EGFR cells, MDA-MB-231 cells, and HCC827 cells, as well as SK-BR-3 cells, but did not kill MCF-7 cells. On the other hand, CAR-T cells transduced with trastuzumab scFv (termed CAR-T-TTZ) efficiently lysed HER2-positive cells, such as MCF-7 HER2 cells, HCC827 cells and SK-BR-3 cells, but not MCF-7 cells or MDA-MB-231 cells (Fig. 1c, d).

**Fig. 2:**
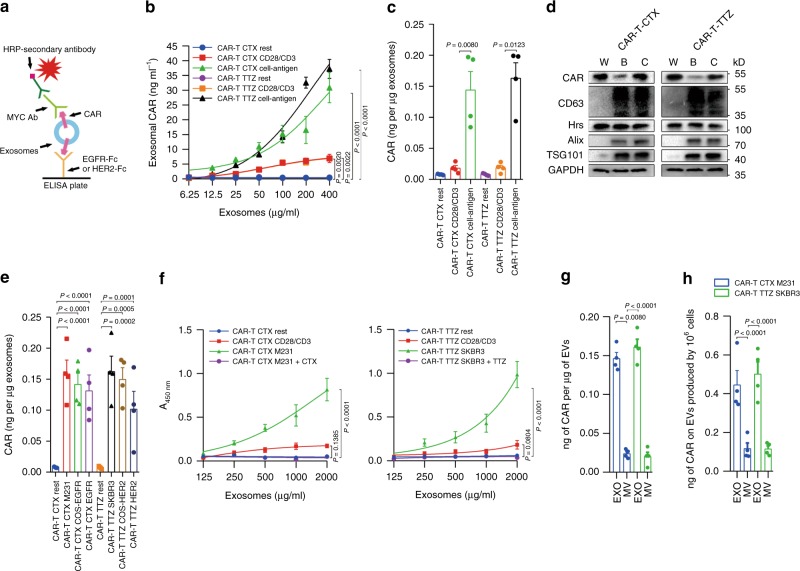
CAR-T cells release extracellular vesicles carrying CAR protein. **a**, **b** Schematic (**a**) of ELISA (**b**) to measure the CAR concentration on the surface of exosomes isolated from CAR-T cells of different states. **c** ELISA of CAR on exosomes from CAR-T, with or without antigen stimulation. **d** Immunoblots for CAR expression in whole-cell lysates (W) and purified exosomes from CAR-T cells with CD28/CD3 bead stimulation (B) or cancer cell stimulation (C). All lanes were loaded with the same amount of total protein. **e** ELISA of CAR on exosomes from CAR-T with or without different stimulation strategies. **f** Antigen binding of exosomes from different cultures with or without blocking antibody cetuximab (CTX) or trastuzumab (TTZ). **g** Levels of CAR on the exosomes or microvesicles derived from CAR-T cells as assayed by ELISA. **h** Levels of exosomal CAR and microvesicle CAR produced by an equal number of CAR-T cells. Results shown represent three (**d**) independent experiments. Data are the means ± s.d. of four independent biological replicates (**b**, **c**, **e**, **f**, **h**). *P* values are from a two-way ANOVA followed by the Bonferroni post-test (**b**, **f**), one-way ANOVA followed by Tukey’s post-test (**c**, **e**) or a two-sided unpaired *t* test (**g**, **h**). Source data (**b**–**h**) are provided as a Source Data file

We stimulated CAR-T-CTX or CAR-T-TTZ cells with a previously described two-stage strategy over the course of 2 weeks in vitro^28^; isolated T cells were first stimulated with anti-CD3/CD28-coated beads. The timing of the second stimulation was based on the return to the resting cell size because cell size is a marker of the lymphocyte activation state, and restimulation of resting lymphocytes reduces activation-induced cell death^29^. Irradiated antigen-expressing cells (MDA-MB-231 cells or SK-BR-3 cells) or anti-CD3/CD28-coated beads were used for the second-stage stimulation, and exosomes were harvested from the culture supernatant using well-established ultracentrifugation protocols^30^. Analysis by enzyme-linked immunosorbent assay (ELISA) and western blotting revealed the presence of CAR expression in exosomes, and its level was significantly higher in exosomes derived from antigen-stimulated CAR-T cells than in those from anti-CD3/CD28 bead-stimulated (Fig. 2a–d). Using different antigen stimulation strategies, such as antigen-expressing COS cells or recombinant antigen-coated beads, also produced a high level of CAR expression in exosomes (Fig. 2e). Iodixanol density gradient centrifugation further confirmed the association of CAR with exosomes (Supplementary Fig. 2a).

T-cell surface CAR can bind to antigen through its extracellular domain to achieve a targeting effect^31^. Using an ELISA and immunoblotting (Fig. 2a–e), we found that exosomal CAR has the same membrane topology as cell surface CAR, with its extracellular domain exposed on the surface of the exosomes. Exosomal CAR binds antigen in a concentration-dependent manner, and this interaction can be disrupted by blocking antibodies (Fig. 2f). CAR was also detected in microvesicles but at a lower level (Fig. 2g, h). Exosomes are generated and released through a defined intracellular trafficking route^32,33^. Genetic knockdown of the ESCRT subunit Hrs, which mediates the recognition and sorting of exosomal cargos^34^, using short hairpin (sh)RNA led to a decreased level of CAR expression in the exosomes (Supplementary Fig. 2b, c).

**Fig. 3:**
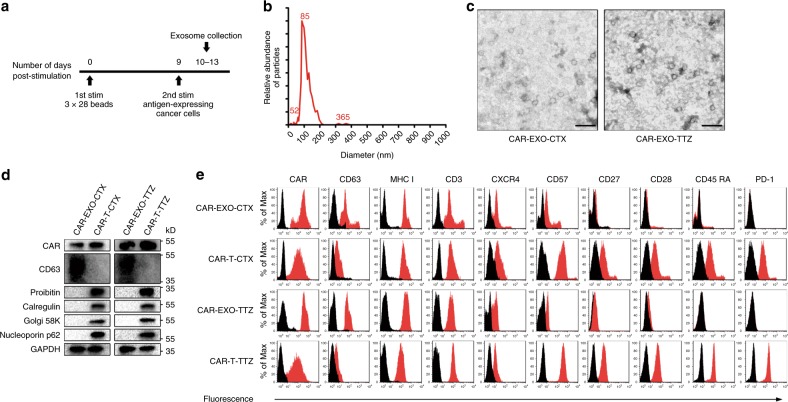
Characterization of CAR exosomes derived from effector CAR-T cells. **a** Schematic representation of the enrichment of CAR-containing exosomes from T cells with repeated antigen stimulation. **b** Size distribution of CAR exosomes as measured by NTA, peaking at an 85-nm diameter. **c** Transmission electron micrographs of CAR exosomes. The samples were negatively stained with uranyl acetate. Scale bars = 100 nm. **d** Immunoblots for CAR expression in exosomes compared with cell markers for endoplasmic reticulum (calregulin), Golgi (Golgi 58 K), mitochondrial (prohibitin), or nuclear (nucleoporin p62) markers. **e** Flow cytometry analyses of CAR exosomes linked to latex beads (4-mm diameter) or CAR-T cells and stained with the indicated primary Abs. The histograms shown in black correspond to the isotype controls of the respective Abs, whereas the red histograms indicate positive fluorescence. Results shown represent three (**b**–**e**) independent experiments. Source data (**d**) are provided as a Source Data file

To further characterize this type of exosome, CAR-containing exosomes were purified using recombinant EGFR- or HER2-coated paramagnetic beads. We obtained 0.1–2 μg of CAR exosomes (based on the protein concentration) per 10^6^ CAR-T cells by repeated antigen stimulation. However, we could not purify sufficient CAR exosomes for the subsequent assays from CAR-T cells with repeated CD3/CD28-bead stimulation. Purified CAR exosomes were termed CAR-EXO-CTX and CAR-EXO-TTZ. CAR exosomes carry surface CAR protein at a level of 0.6 ng per μg of exosomes, as determined by ELISA, which is comparable to the expression level of CARs in CAR-T cells (Supplementary Fig. 3). The exosomes produced were physically homogeneous, with a size distribution peaking at an 80-nm diameter, as determined by nanoparticle tracking analysis (NTA) and electron microscopy (Fig. 3a–c). Further characterization of T-cell exosomes by western blot analysis confirmed the presence of typical exosomal proteins after purification but also excluded the presence of contaminating proteins during purification from the endoplasmic reticulum, Golgi, mitochondria, and nucleus (i.e., the calregulin, Golgi 58K, prohibitin, and nucleoporin molecules, respectively), potentially derived from apoptotic or dead cells, compared with cell lysates (Fig. 3d). These results were consistent with those obtained using flow cytometry analysis after exosome coupling to latex beads^35^. In fact, CAR-containing exosomes expressed appreciable levels of both CAR and CD63, further confirming their exosomal nature, as well as MHC I proteins and a substantial amount of CD3, CXCR4, and CD57, whereas CD27 receptor and CD28 were expressed at relatively low levels, and CD45 RA and PD-1 receptor were undetectable (Fig. 3e and Supplementary Fig. 1).

### Cytolytic activity of CAR exosomes

**Fig. 4:**
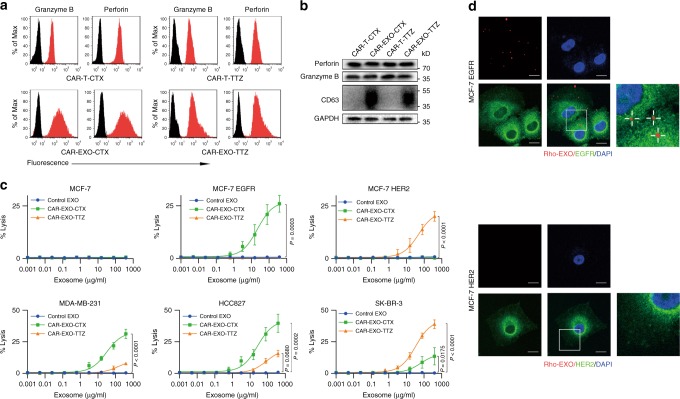
Cytolytic activity of CAR exosomes in vitro. **a** Flow cytometry analyses of CAR exosomes linked to latex beads (4-mm diameter) or CAR-T cells stained with the indicated primary Abs. The histograms shown in black correspond to the isotype controls of the respective Abs, whereas the red histograms indicate positive fluorescence. **b** Immunoblots for perforin and granzyme B expression in CAR exosomes and CAR-T cells. **c** Killing activity of CAR exosomes in response to tumour cells. The cytotoxic activity of CAR exosomes and control T cells against cancer cell lines was assessed by the ^51^Cr-release assay at the indicated concentration. **d** Confocal microscopy analysis of MCF-7 EGFR cells (up) and MCF-7 HER2 cells (down) after incubation with NHS-Rhodamine (Rho)-labelled CAR-EXO-CTX for 2 h. The experiments were repeated independently three times with similar results. Scale bars = 10 μm. Results shown represent three (**a**, **b**, **d**) independent experiments. Data are the means ± s.d. of three (**c**) independent biological replicates. *P* values were from a nonparametric *t* test (**c**). Source data (**b**, **c**) are provided as a Source Data file

CTL and NK cells are known to exert their cytolytic activity through the release of cytotoxic effectors (i.e., granzyme B and perforin) contained in lytic granules. Upon target cell recognition and conjugation, the granules are actively directed to the site of cell−cell contact, and soluble effector molecules are released into the forming cytotoxic immunological synapse. Thus, we first evaluated the presence of granzyme B and perforin on CAR exosomes by flow cytometry analyses of exosome-bead complexes. The results showed that perforin and granzyme B molecules were notably expressed on both CAR-EXO-CTX and CAR-EXO-TTZ (Fig. 4a and Supplementary Fig. 1). Further western blot analysis revealed the presence of both granzyme B and perforin in CAR-T cells and CAR exosomes (Fig. 4b).

To test the potential bioactivity of granzyme B and perforin expressed by CAR exosomes, we investigated their cytotoxic effect against human tumour cell lines and exosomes from non-transduced T cells, which served as the control (Fig. 4c). In a panel of cell lines, CAR-EXO-CTX and CAR-EXO-TTZ show notable cytotoxic effects on EGFR-expressing cells or HER2-expressing cells, while cancer cells with low antigen expression seemed to be resistant to exosome-mediated lysis (Supplementary Table 1). These data suggested that CAR exosomes can exert strong and specific cytotoxic activity against cancer cells. Exosomes may undergo uptake by tumour cells through a fusion-mediated mechanism^36^. Figure 4d shows that CAR-EXO-CTX, labelled with NHS-Rhodamine dye (red), was detected inside the MCF-7 EGFR cells after 2 h of incubation. No evidence of CAR-EXO-CTX uptake was obtained when MCF-7 HER2 cells were used as target cells.

### CAR exosomes have potent antitumour activity in vivo

Having demonstrated both the specificity and cytotoxicity of CAR exosomes in vitro, we sought to confirm the in vivo antitumour activity of these exosomes. To support the idea that CAR exosomes have no undesired toxicity, in our study, we conducted a 13-week repeat-dose toxicity study with a 4-week recovery period. The maximum tolerated dose (MTD) was defined as the dose at which no deaths occurred, and the body weight loss was ≤20% of the animal weight pretreatment. In this study, animals injected with CAR exosomes exhibited no signs of toxicity, even at the highest dose tested (Supplementary Fig. 4). Thus, the MTD of the CAR exosomes was not reached in this study.

**Fig. 5:**
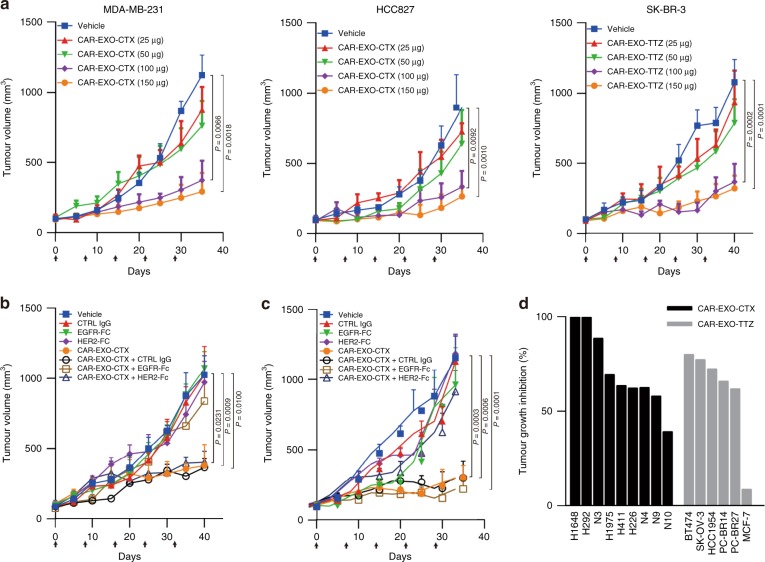
CAR exosomes have notable antitumour activity in vivo. **a** Tumour volumes of MDA-MB-231 (left), HCC827 (middle) and SK-BR-3 (right) tumour xenografts after treatment with the indicated treatment, *n* = 8. **b**, **c** Tumour volumes of MDA-MB-231 (**b**) and SK-BR-3 (**c**) tumour xenografts after treatment with the indicated CAR exosome treatment with or without blocking recombinant antigen, *n* = 8. **d** Cancer cell lines or patient-derived tumour tissue fragments established as subcutaneous xenografts (*n* = 8) and treated with weekly doses of CAR exosomes (100 μg). Substantial TGI was observed in lung cancer models treated with CAR-EXO-CTX (black bars) and in HER2-positive breast and ovary cancer models treated with CAR-EXO-TTZ (grey bars). Arrows indicate the treatment point (**a**–**c**). Data are means ± s.e.m. (**a**–**c**). *P* values are from a two-way ANOVA followed by Bonferroni post-test (**a**–**c**). Source data (**a**–**d**) are provided as a Source Data file

CAR-EXO-CTX showed dose-dependent tumour growth inhibition (TGI) in both MDA-MB-231 and HCC827 mouse xenograft models (Fig. 5a). Intravenous doses of 100−150 μg of CAR-EXO-CTX were notably efficacious, achieving more than 70% TGI compared with the controls. Only partial TGI was observed with 25 to 50 μg of CAR-EXO-CTX, indicating suboptimal dosing. In a HER2^+^ cell line-based mouse xenograft model, 100−150 μg of CAR-EXO-TTZ treatment also showed a marked antitumour effect with approximately 67% TGI. To further examine the specificity, we further characterized the antitumour effect of CAR exosomes using different recombinant antigens in vivo. Our data showed that the injection of CAR-EXO-CTX notably inhibited the growth of tumours derived from MDA-MB-231 cells, whereas combined treatment of the exosomes with EGFR-Fc protein antibodies, but not with IgG isotype antibodies or HER2-Fc protein, reversed the effect (Fig. 5b). Moreover, in the SK-BR-3 xenograft model, although CAR-EXO-TTZ notably inhibited tumour growth, this effect was weakened by combining exosomes with the HER2-Fc protein (Fig. 5c).

Additionally, we observed that the antitumour efficacy of single-agent CAR exosomes induced potent TGI in EGFR- or HER2-positive human cancer cell line models or patient-derived xenograft models (Fig. 5d and Supplementary Fig. 5). Treatment with 4−5 cycles of weekly exosome treatment at doses of 100 μg resulted in TGI in all five EGFR-positive NSCLC models and four PDX models. Substantial TGI (>50%) was also observed in three HER2^+^ cancer cell line xenograft models and two PDX models after CAR-EXO-TTZ treatment.

### CAR exosomes do not express PD-1

**Fig. 6:**
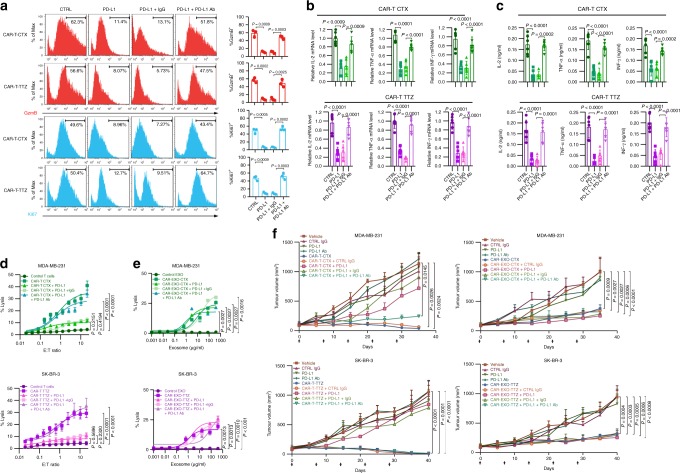
PD-L1 inhibits CAR-T cells but not CAR exosomes in vitro and in vivo. **a** Representative histogram of the expression of granzyme B (GzmB, red) and Ki-67 (blue) after the indicated treatments in CAR-T cells. The proportions of cells with positive GzmB or Ki-67 expression are shown on the right. **b** qPCR analyses of IL-2, IFN-γ, and TNF in CAR-T cells after the indicated treatments with or without blocking by recombinant PD-L1 or the anti-PD-L1 antibodies. The relative mRNA expression level was calculated as the ratio to the control cells. **c** ELISA of IL-2, IFN-γ, and TNF in CAR-T cells after the indicated treatment with or without blocking by recombinant PD-L1 or the anti-PD-L1 antibodies. **d**, **e** Killing activity of CAR-T cells (**d**) and CAR exosomes (**e**) in response to tumour cells. The cytotoxic activity of CAR-T or CAR exosomes against cancer cell lines was assessed by the ^51^Cr-release assay at the indicated effector-to-target (E:T) ratios or indicated concentrations with or without blocking by recombinant PD-L1 or anti-PD-L1 antibodies. **f** Tumour volumes of MDA-MB-231 (up) and SK-BR-3 (down) tumour xenografts after the indicated treatment (left, CAR-T cells; right CAR exosomes). *n* = 8. Arrows indicate the treatment point (**f**). Data are mean ± s.d. (**a**–**e**) of three (**a**) or six (**b**–**e**) independent biological replicates or means ± s.e.m. (**e**). *P* values are from a two-sided unpaired *t* test (**a**–**c**) or a two-way ANOVA followed by the Bonferroni post-test (**d**–**f**); Source data (**a**–**f**) are provided as a Source Data file

Because human CAR-T cells may be reversibly inactivated within the solid tumour microenvironment of some tumours via multiple mechanisms, such as the PD-1 pathway, we next sought to investigate whether CAR exosomes may be inactivated for the same reason. The current model for PD-L1-mediated immunosuppression is based on the interaction between PD-L1 on the tumour cell surface and PD-1 on T cells. Recombinant PD-L1 protein treatment resulted in decreased proliferation and decreased IFN-γ, IL-10, IL-4, and IL-2 secretion from anti-CD3-stimulated T cells in vitro^37,38^. Moreover, recombinant PD-L1 was reported to promote in vivo cardiac allograft survival and protection from chronic rejection^39^, long-term pancreas islet allograft survival^40^, and protection of mouse survival in models of colitis^41^. Therefore, we first tested whether PD-L1 inhibits CAR-T cells. Recombinant PD-L1 treatment notably inhibited the proliferation, cytokine production and cytotoxicity of CAR-T cells, as demonstrated by the reduced expression of Ki-67 and granzyme B (GzmB) in CAR-T cells and the inhibited production of IFN-γ, IL-2, and TNF (Fig. 6a–c and Supplementary Fig. 1). Combined treatment with the recombinant PD-L1 and anti-PD-L1 antibodies nearly abolished these effects. Moreover, pre-treating CAR-T cells with recombinant PD-L1 inhibited their ability to kill their target cells (Fig. 6d-e). Conversely, adding recombinant PD-L1 to CAR exosomes did not cause significant loss of cytolytic activity in cell line assays; the cause may be the leak of PD-1 expression of CAR exosomes. We next examined the effects of PD-L1 on CAR-T cells or CAR exosomes in vivo, and injection of CAR exosomes notably inhibited the growth of tumours derived from MDA-MB-231 cells or SK-BR-3 cells, with or without combined PD-L1 treatment, whereas combined treatment of the CAR-T cells with the PD-L1 protein, but not with IgG isotype antibodies, inhibited the antitumour effect (Fig. 6f). Similar results were also observed when CAR exosomes or CAR-T cells were administered via the intratumoural (i.t.) route (Supplementary Fig. 6). These data suggest that PD-L1 suppresses the antitumour immunity of CAR-T cells but not exosomes.

### No observation of CRS after CAR exosome immunotherapy

**Fig. 7:**
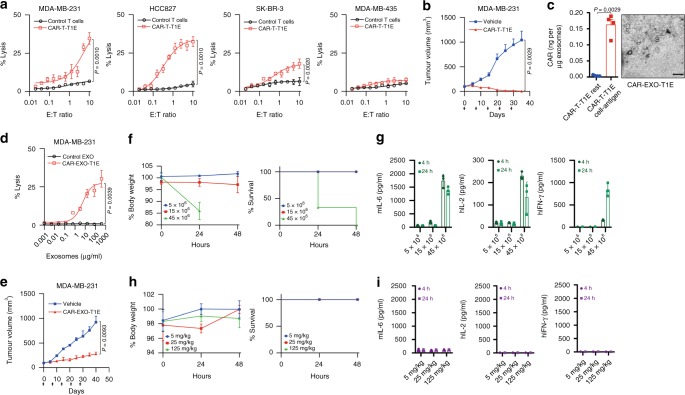
CAR exosomes do not cause cytokine release syndrome in mice. **a** Killing activity of CAR-T-T1E cells in response to tumour cells. The cytotoxic activity of CAR-T-T1E and control T cells against cancer cell lines was assessed by the ^51^Cr-release assay at the indicated effector-to-target (E:T) ratios. **b** Tumour volumes of MDA-MB-231 tumour xenografts after the indicated treatment, *n* = 8. **c** ELISA (left) of CAR on exosomes from CAR-T-T1E and transmission electron micrographs of CAR-EXO-T1E (right). Scale bars = 100 nm. **d** Cytotoxic activity of CAR exosomes and control T cells against cancer cell lines was assessed by the ^51^Cr-release assay at the indicated concentration. **e** Tumour volumes of MDA-MB-231 tumour xenografts after the indicated treatment, *n* = 8. Arrows indicate the treatment point (**e**). **f** Three groups of three tumour-free mice were treated i.p. with CAR-T-T1E cells at the indicated escalating doses. Serial weight measurements were performed and normalized to the starting body weight for each animal. The Kaplan–Meier survival curve of mice in all three groups is shown on the right. **g** Serum levels of human (h) and mouse (m) cytokines, measured at the indicated time points. **h** Three groups of three tumour-free mice were treated i.p. with CAR-EXO-T1E cells at the indicated escalating doses. Serial weight measurements were performed and normalized to the starting body weight for each animal. The Kaplan–Meier survival curve of mice in all three groups is shown on the right. **i** Serum levels of human (h) and mouse (m) cytokines, measured at the indicated time points. Arrows (**b**, **e**) indicate the treatment point. Results shown represent three (**c**) independent experiments. The data are presented as the means ± s.d. of six (**a**); four (**c**) or three (**f**–**i**) independent biological replicates or means ± s.e.m. three (**f**–**i**) independent biological replicates. *P* values are from a nonparametric *t* test (**a**, **b**, **d**, **e**) or a two-sided unpaired *t* test (**c**). Source data (**a**–**i**) are provided as a Source Data file

A previous report showed that T cells engineered with a broad-ErbB dimer targeting CAR, T1E28z, recognize both human and mouse ErbB^+^ cells and show dose-dependent side effects similar to CRS in mice when delivered intra-peritoneally (i.p.)^42,43^. We next sought to determine whether exosomes may ameliorate CRS risk in similar models. We fused a T1E sequence with a CD8a hinge and transmembrane domain and the intracellular domains of human 4-1BB and CD3ζ similar to CAR-T-CTX and CAR-T-TTZ, and this construct was termed CAR-T-T1E (Supplementary Figs. 1 and 7). Consistent with a previous report^42^, cocultivation experiments demonstrated that CAR-T-T1E cell recognition of cancer cells expressed all possible EGFR- or HER3-based dimers, leading to the production of IL-2, TNF and IFN-γ, whereas CAR-T-CTX and CAR-T-TTZ were only activated by the corresponding antigen-expressing cells (Supplementary Fig. 8). Moreover, CAR-T-T1E efficiently lysed MDA-MB-231 cells and HCC827 cells but exerted a weaker effect on SK-BR-3 cells and MDA-MB-435 cells (Fig. 7a). CAR-T-T1E also exerted a striking antitumour effect on MDA-MB-231 cell-based xenografts (Fig. 7b). Next, CAR-T1E exosomes were detected by ELISA and purified using methods similar to those used in our previous experiments, and they were termed CAR-EXO-T1E (Fig. 7c). As expected, CAR-EXO-T1E also show cytolytic activity in MDA-MB-231 cell monolayers and antitumour effects on MDA-MB-231 xenografts (Fig. 7d, e).

It was reported that T1E-CAR-containing T cells can cause dose-dependent toxicity similar to CRS when delivered i.p. in SCID beige mice^43^. To confirm this result in our experiments, groups of mice were treated with escalating doses of CAR-T-T1E immunotherapy, administered i.p. Because there was a risk of severe toxicity induction, only three mice were included in each group for ethical reasons. Animals that received 5 or 15 million cells exhibited no alteration in behaviour or weight during the ensuing 48 h. In contrast, animals that received 45 million cells demonstrated subdued behaviour, piloerection, and reduced mobility within 24 h, accompanied by rapid weight loss (Fig. 7f), and all animals died within 48 h. Serial blood samples revealed that human IFN-γ, human IL-2, and mouse IL-6 (Fig. 7g) were all detectable in the circulation of mice that had received a lethal dose of CAR-T cells. Next, we examined whether CAR-EXO-T1E has a similar risk of cytokine release in response to immunotherapy. Different doses of CAR-EXO-T1E were administered by i.p. in mice. In all groups of mice, no alteration in behaviour or weight gain was observed, and human cytokines were not detected in the circulation of these mice (Fig. 7h, i).

## Discussion

CAR-based adoptive immunotherapies, which use genetically modified T lymphocytes to provide both tumour targeting and immune responses, can act as living drugs that exert constant cytotoxic attacks on targeted cells. The tumour-killing capacity of CAR-T cells relies on the life span of the cells themselves and on further in vivo replication. However, CAR-T cells and the replication of these cells in vivo can boost cytokine release, which is not suitably controllable. This effect is a potential source of adverse effects, such as CRS, the cytokine storm and on-target, off-tumour responses. In solid tumours, CAR-T therapy has not achieved the clinical success that has been observed in haematological malignancies. One reason for the poor treatment response is the failure of CAR-T cells to accumulate and replicate in hostile tumour microenvironments^44,45^. Moreover, CAR-T-cell inactivation and possible exclusion from the tumour mass, reciprocal interactions between stromal cells and tumours, and the propensity of cancers such as prostate cancer to disseminate preferentially to bones may all be due to the absence of a substantial CAR-mediated T-cell response in solid tumours^46–48^.

In this report, we show that CAR exosomes, which are released from CAR-T cells, also hold great therapeutic potential for attacking cancer cells. Using exosomes as direct attackers for cancer therapy may have several obvious advantages. CAR exosomes may have a low risk of toxicities, such as CRS, and CAR exosomes can be generated from healthy donors and therefore have the potential to be an ‘off‑the‑shelf therapeutic’. The manufacturing process of the cell-free vesicles is also safer than that of living CAR-T cells, supported by a recent case report showing that the CAR gene was unintentionally introduced into a single leukaemic B cell during T-cell manufacturing and that its product bound in *cis* to the CD19 epitope on the surface of leukaemic cells, masking it from recognition by and conferring resistance to CTL019; the patient ultimately died of complications related to progressive leukaemia^49^. Second, because exosomes have a nanoscale size, they have the advantage of being utilized for solid tumour therapy. The appropriate use of exosomes paves the way to targeting tumour cells in specific regions, such as glioblastomas^25,50^. Third, the combined and/or alternate utilization of these two platforms (i.e., CAR-T cells and CAR exosomes) will undoubtedly strengthen the use of CAR-based cancer therapy; our data show that eventually, PD-L1 treatment did not impede exosome cytotoxicity, suggesting that CAR exosome treatment can overcome immunosuppressive mechanisms^17^. For the clinical application of CAR exosomes, the proposed scheme includes T-cell isolation from the peripheral blood of healthy donors or cancer patients, genetic engineering of T cells to express CARs, in vitro replication and stimulation of CAR-engineered T cells, isolation of exosomes, purification of CAR exosomes, and exosome infusion into patients. All the reported and standard approaches can be used to isolate exosomes from culture media, including ultrafiltration^51^, ultracentrifugation^30^ and affinity capture on magnetic beads^52^. CAR exosomes can be further purified by antigen-coated magnetic beads as used in our study.

Because of the different natural properties between CAR-T cells and CAR exosomes, we cannot simply compare the kill efficiency. Our data show that 5 × 10^4^ CAR-T cells or 10 μg CAR exosomes can cause approximately 20% killing of 5000 tumour cells. However, unlike cell-based treatment, the exact number of exosomes present in 1 µg of protein is controversial based on current methods^53^. We used ELISA to quantify CAR expression in CAR-T cells and CAR exosomes. There are approximately 0.25−0.69 ng CAR protein per μg CAR-T cells based on the protein concentration, corresponding to approximately 10 ng CAR protein per 5 × 10^4^ CAR-T cells, while there are approximately 0.6 ng CAR protein per 1 μg CAR exosomes (6 ng per 10 μg CAR exosomes). The CAR protein level is very comparable between CAR-T cells and CAR exosomes used to achieve similar cell lysis effects in vitro. The present study has limitations. We provide evidence that the targeting effect and antitumour effect with CAR exosomes can be achieved, but whether CAR signalling or the granzymes are required for the action mode is unknown. Moreover, the precise mechanisms responsible for these therapeutic effects are currently not well characterized. Hence, these findings will need further validation.

To conclude, engineered T-cell adoptive immunotherapy is a promising therapy for cancer treatment. This therapy also has some limitations or defects in the context of the volatility and complexity of cancer. Our data showed that CAR exosomes can act as eventual attackers, thereby conquering some of the limitations of the current treatment models. Applying appropriately both cellular and exosomal platforms, CAR-based treatment will be more effective and might be the next promising option for targeted cancer treatment.

## Methods

### Cell lines, antibodies and recombinant proteins

All cell lines were purchased from the American Type Culture Collection (ATCC, Manassas, VA). The identities of the cell lines were verified by STR analysis, and the cell lines were confirmed to be mycoplasma free. The cells were maintained in DMEM with 10% foetal bovine serum. Cell culture media and supplements were obtained from Life Technologies, Inc. Recombinant human PD-L1/B7-H1 Fc chimaera protein was obtained from R&D Systems (Minneapolis, MN). Cetuximab was purchased from Merck. Trastuzumab and atezolizumab were purchased from Roche Ltd. Residues 1–619 of the extracellular domain of EGFR (EGFR-ECD) and residues 1–646 of HER2-ECD were prepared using the pcDNA3.4 expression vector (Invitrogen) and FreeStyle 293 expression system (Invitrogen)^54,55^. Information about the primary antibodies used in this work is included in Supplementary Table 2.

### Vector construction

The sequence encoding the scFv antibodies generated from cetuximab or trastuzumab was chemically synthesized. As the targeting of HER2 with high affinity CAR-T cells was reported to lead to serious toxicity due to target recognition on normal cardiopulmonary tissue^56^, the 4D5-5 scFv derived from trastuzumab was chosen for this study, which exhibited robust antitumour efficacy against HER2-overexpressing cancer cells both in vitro and in xenogeneic mouse tumour models but spared normal cells expressing physiologic target levels^57,58^. As shown in Fig. 1a, CAR-CTX and CAR-TTZ contained the human CD8α signal peptide followed by the scFv linked in-frame to the hinge domain of the CD8α molecule, transmembrane region of the human CD8 molecule, and intracellular signalling domains of the CD137 and CD3ζ molecules. Similar construction methods were further used for CAR-T1E^42^. The fragments were subcloned into the pELNS vector^59^. High-titre replication-defective lentiviral vectors were produced and concentrated^59^.

### In vitro T-cell transduction and cultures

Negative selection using RosetteSep kits (Stem Cell Technologies) was adopted to isolate primary human T cells from healthy volunteer donors following leukapheresis. All specimens were collected under an approved protocol by the Second Military Medical University Review Board, and written informed consent was obtained from each donor. Dynabeads Human T-Activator CD3/CD28 (Life Technologies) used to stimulate T cells was used at a bead-to-cell ratio of 3:1 (first stimulation). T cells were cultured in RPMI 1640 medium supplemented with 10% foetal calf serum, HEPES buffer (20 mM), and penicillin and streptomycin (1%). The timing of the second stimulation was based on the return to resting cell size because cell size is a marker of the lymphocyte activation state, and restimulation of resting lymphocytes reduces activation-induced cell death^29^. The end of stimulation was determined based on a decrease in log-phase growth and reduction in the mean lymphocytic volume to 300–330 fl, as measured on a Coulter Multisizer. This stage is usually reached 9−10 days after stimulation, at which point the cells are frozen for in vitro/in vivo functional assays or are used for restimulation and purification of exosomes. T cells from the first stimulation were normalized to 30% CAR^+^ and were cocultured with either antigen-expressing cancer cells or Dynabeads Human T-Activator CD3/CD28 at a 1:1 ratio (second stimulation). Antigen-expressing cancer cells were irradiated with 100 Gy of γ-radiation and washed before co-culture. In the second stimulation period, IL-2 (120 IU/ml) was added every other day.

### Cytotoxicity assays

Cytotoxicity of the CAR-expressing T cells or exosomes was tested in a standard 4-h ^51^Cr-release assay^28^. Target cells were labelled with ^51^Cr for 1 h at 37 °C. Radioactive ^51^Cr (50 μCi) was used to label 1 × 10^6^ target cells. One hundred microliters of labelled target cells (*n* = 5000) as plated in each well of a 96-well plate. Effector cells were added at a volume of 100 μl at different E:T ratios. Exosomes were added at different concentrations. CAR-T cells or exosomes and targets were incubated together for 4 h at 37 °C. The supernatant (30 μl) from each well was collected and transferred to the filter of a LumaPlate. The filter was allowed to dry overnight. Radioactivity released into the culture medium was measured using a β-emission-reading liquid scintillation counter. The percentage of specific lysis was calculated as follows: (sample counts – spontaneous counts)/(maximum counts – spontaneous counts) × 100.

### Isolation and purification of exosomes

Exosome purification from cell culture supernatants^60^. Briefly, culture supernatants were centrifuged at 300 *×* *g* for 5 min and then at 1200 *×* *g* for 20 min and 10,000 *×* *g* for 30 min to eliminate cells and debris. The supernatants were ultracentrifuged at 100,000 *×* *g* for 60 min at 4 °C to pellet exosomes. The exosome pellets were washed twice in a large volume of PBS and were recovered by centrifugation at 100,000 *×* *g* for 1 h. Exosomal protein was measured by the Bradford assay with the Bio-Rad Protein Assay Reagent (Bio-Rad, Hemel Hempstead, UK) and stored at −80 °C.

For further purification of CAR exosomes, those isolated from CAR-T cell culture supernatants were resuspended in PBS. The supernatants were then mixed with EGFR- or HER2-coated Dynabeads (Dynal Biotech, Oslo, Norway). The mixture was incubated overnight at 4 °C on a rotating plate, and the beads were collected and washed twice with PBS on a magnetic rack to eliminate unbound or excess exosomes. CAR exosomes were eluted with 0.1 M sodium citrate/citric acid and were immediately equilibrated at the desired pH.

### ELISA

To detect CAR expression on EVs or cell supernatants, ELISA plates (96-well) (Biolegend) were coated with 0.25 μg per well (100 μl) of recombinant antigen (EGFR or HER2) overnight at 4 °C. Free binding sites were blocked with 200 μl of blocking buffer (Pierce) for 1 h at room temperature. Next, extracellular vesicle samples purified from cell culture supernatants were added to each well. The exosome or microvesicle samples purified from cell culture supernatants were prepared by serial dilution according to the total protein level to analyse the expression of CAR on exosomes and microvesicles. The concentration of CAR on the surface of exosomes isolated from the indicated cells was calculated based on the linear range of the ELISA data. After overnight incubation at 4 °C, MYC Tag Monoclonal Antibody (R950-25, Invitrogen) was added to each well and incubated for 1 h at room temperature. A total of 100 μl per well of horseradish peroxidase-conjugated secondary antibody (BD Biosciences) diluted in PBS containing 0.1% BSA was then added and incubated for 1 h at room temperature. The plates were developed with tetramethylbenzidine (Pierce), and the reactions were stopped with 0.5 N H_2_SO_4_. The plates were read at 450 nm using a BioTek plate reader. Recombinant MYC-tagged scFv protein was used to make a standard curve. Recombinant P-selectin protein (R&D Systems; Cat# 137-PS) was used as a negative control to verify the detection specificity. The results of the standard curve demonstrated that the established ELISA exhibited a reliable linear detection range from 0.2 to 25 ng/ml. For the detection of IFN-γ, TNF and IL-2, the supernatant of CAR-T cells was harvested and measured according to the manufacturer’s instructions (Biolegend).

### Exosomal binding assay

To test the binding of exosomal CAR to antigen, 100 μl of exosome samples of different concentrations was captured onto recombinant antigen (EGFR or HER2)-coated 96-well ELISA plates by overnight incubation at 4 °C. Next, 100 μl of 4 μg/ml anti-MYC antibody was added and incubated for 2 h at room temperature. Cetuximab and trastuzumab were added as indicated, at a concentration of 10 μg/ml. A total of 100 μl per well of horseradish peroxidase-conjugated secondary antibody (BD Biosciences) diluted in PBS containing 0.1% BSA was then added and incubated for 1 h at room temperature. The plates were developed with tetramethylbenzidine (Pierce), and the reactions were stopped using 0.5 N H_2_SO_4_. The plates were read at 450 nm using a BioTek plate reader. Recombinant MYC-tagged scFv protein directly coated onto the plates was used as the positive control.

### Generation of stable Hrs-knockdown T cells

Short hairpin RNAs (shRNAs) against human Hrs (also known as HGS) (NM_004712, GCACGTCTTTCCAGAATTCAA, GCATGAAGAGTAACCACAGC), or scrambled shRNA-control (Addgene) were packaged into lentiviral particles using 293T cells co-transfected with the viral packaging plasmids. Lentiviral supernatants were harvested 48–72 h after transfection. Cells were infected with filtered lentivirus and selected by 2 μg/ml puromycin.

### Exosome characterization

For the verification of purified exosomes using electron microscopy, purified exosomes suspended in PBS were dropped on formvar carbon-coated nickel grids. After staining with 2% uranyl acetate, the grids were air-dried and visualized using an H-7650 transmission electron microscope (TEM, Hitachi, Japan). The size and concentration of CAR exosomes were determined using a NanoSight NS300 (Malvern Instruments), which is equipped with fast video capture and particle-tracking software. For iodixanol density gradient centrifugation, exosomes harvested by differential centrifugation were loaded on top of a discontinuous iodixanol gradient (5, 10, 20 and 40%, made by diluting 60% OptiPrep aqueous iodixanol with 0.25 M sucrose in 10 mM Tris) and centrifuged at 100,000 *×* *g* for 18 h at 4 °C (Beckman Coulter, Optima MAX-XP). Ten fractions of equal volume were collected from the top of the gradients, with the exosomes distributed in the density range between 1.13 and 1.19 g/ml, as previously demonstrated^30,61–63^. The exosomes were further pelleted by ultracentrifugation at 100,000 *×* *g* for 4 h at 4 °C.

### Flow cytometry

Purified exosomes were incubated with 4-μm-diameter aldehyde/sulphate latex beads (Interfacial Dynamics) in PBS overnight at 4 °C under gentle agitation. Exosome or cell surface staining was performed for 30 min at 4 °C and was analysed using a FACSCalibur flow cytometer (BD Biosciences) and CellQuest Software (BD Biosciences). Intra-exosome or cellular staining was performed for 60 min on ice after using a fixation/permeabilization kit (eBioscience). A minimum of 1 × 10^4^ beads/sample was examined.

### Immunofluorescence staining

To verify the physical interactions between exosomes and cancer cells, purified exosomes were stained with NHS-Rhodamine (Pierce) in 100 μl PBS, washed with 10 ml PBS and pelleted by ultracentrifugation. MCF7-EGFR or MCF-HER2 cells were treated with Rho-labelled exosomes (25 μg/ml) for 2 h and then fixed for confocal microscopy after immunostaining for cancer cells. For immunofluorescence staining, the cells were plated on cover slides, fixed with 4% paraformaldehyde, permeabilized with 0.3% Triton X-100, and blocked with 1% BSA. The primary antibodies were incubated overnight and subjected to the corresponding fluorescent dye-conjugated secondary antibody for labelling. Nuclei were stained with DAPI. The samples were observed, and pictures were taken using a Leica TCS SP2 confocal system (Leica, Germany).

### Western blot analysis

Whole-cell lysates or exosomal proteins were separated using SDS–PAGE and transferred onto PVDF membranes. The blots were blocked with 10% non-fat dry milk at room temperature for 1 h and were incubated overnight at 4 °C with the corresponding primary antibodies at dilutions recommended by the suppliers, followed by incubation with HRP-conjugated secondary antibodies (Cell Signalling Technology) at room temperature for 1 h. The blots on the membranes were developed using ECL detection reagents (Pierce). CD63, Hrs, Alix, and TSG101 were used as exosome markers. GAPDH was used as a loading control.

### Repeat-dose toxicity studies

A 13-week repeat-dose toxicity study with a 4-week recovery period was conducted to evaluate the safety of all drugs used in the in vivo experiments. Groups of SCID mice were dosed with 2 × 10^5^, 1 × 10^6^, 5 × 10^6^, or 1 × 10^7^ CAR-T cells or 25, 50, 100 or 150 μg of exosomes once weekly. Mice were monitored for visible signs of toxicity and body weight loss. The MTD was defined as the dose at which no deaths occurred and the body weight loss was ≤20% of the pretreatment animal weight. Upon termination of the study, the animals were sacrificed, and tissue from the selected mice was harvested and subjected to histopathologic analysis.

### Quantitative PCR (qPCR)

Total RNA was isolated using the RNeasy Mini Kit (Qiagen), according to the manufacturer’s specifications. Real-time quantitative PCR was performed on an ABI PRISM 7900HT and analysed using the SDSv2.3 (Applied Biosystems). *GAPDH* was used as an internal control. Information about the primers is included in Supplementary Table 3.

### In vivo study

In vivo experiments were approved by the Institutional Animal Care and Use Committee (IACUC) of Second Military Medical University, and mice were housed in a specific pathogen-free barrier facility. Cancer cells were inoculated into BALB/c nude mice (Shanghai Experimental Animal Centre of Chinese Academy of Sciences). When the tumour volumes reached an average of approximately 100 mm^3^, the mice were randomly divided into groups of 6−10 mice each. The mice were injected i.p. with 200 mg/kg cyclophosphamide to deplete the host lymphocyte compartments. For patient-derived xenograft models, patient specimens were obtained during initial surgery from primary-diagnosed, early-stage cancer patients at Changhai Hospital of Second Military Medical University. Written informed consent was obtained from each patient, and the local ethics committee of Changhai Hospital, Second Military Medical University approved experimental design and tissue samples collection. Tumour pathology was evaluated by hospital pathologists. Minimally passaged human tumour xenograft models and tumour-initiating cell frequency were established^54,64^. Briefly, human tumour xenograft models were established by subcutaneous implantation of patient-derived solid tissue fragments in NOD/SCID mice (Animal Centre of Chinese Academy of Sciences). All animals were treated in accordance with the guidelines of the Committee on Animals of the Second Military Medical University. The growing tumours were allowed to engraft to approximately 100 mm^3^ prior to treatment. Multiple-dose studies consisted of weekly treatment. The tumours were measured with digital callipers, and the tumour volumes were calculated by the following formula: volume = length × (width)^2^/2. For cell treatment, CAR-T cells or exosomes were i.v. or i.t. injected into mice every week for the entire course of treatment with the indicated dose. Mice were i.p. injected with 2000 units of IL-2 twice a week following infusion. Mice were killed 40 days after treatment or if the volume of the tumours reached 1300 mm^2^ cm before 35 days.

Recombinant PD-L1 or PD-L1 Ab was used when necessary at a dose of 10 mg/kg very week. The percentage TGI compared with controls was calculated at the end of the study when indicated.

### Statistical analysis

Unless otherwise specified, Student’s *t* test was used to evaluate the significance of differences between two groups, and ANOVA was used to evaluate differences among three or more groups. Differences between samples were considered statistically significant when *P* < 0.05.

### Reporting summary

Further information on research design is available in the Nature Research Reporting Summary linked to this article.

## Supplementary information

Supplementary Information

Reporting Summary

Source Data

## Data Availability

The authors declare that the data supporting the findings of this study are available within the paper and its Supplementary Information files or from the corresponding author on reasonable request. The source data underlying Figs. 1c, 2b–d, 3d, 4b, c, 5a−d, 6a−f and 7a−i and Supplementary Figs. 2a−c, 3a−c, 4a, b, 5, 6 and 8a−c are provided as a Source Data file.
