# Supplementary material for: CAR exosomes derived from effector CAR-T cells have potent antitumour effects and low toxicity
Source: Nat Commun. 2019 Sep 25;10:4355. doi: 10.1038/s41467-019-12321-3 (PMC6761190; doi:10.1038/s41467-019-12321-3)
Supplement: Supplementary file 1 — Supplementary Information [file 41467_2019_12321_MOESM1_ESM.pdf]

Supplementary Information for

CAR Exosomes Derived from Effector CAR-T cells Have Potent Antitumour Effects and Low Toxicity

Fu *et al*

Supplementary Figures

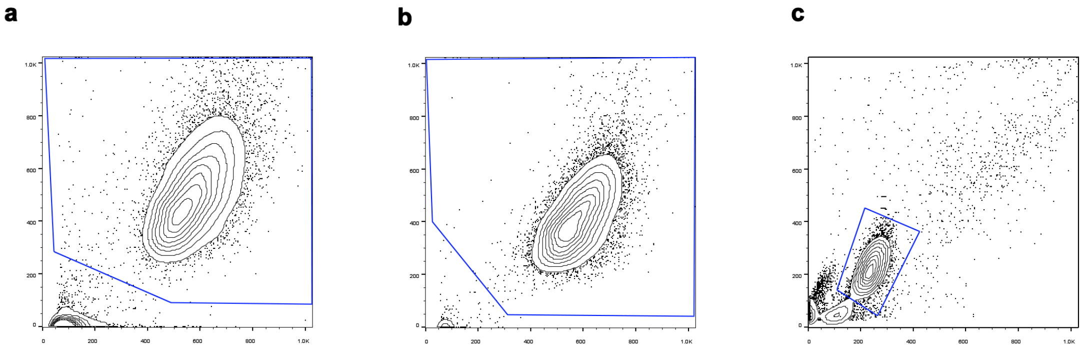

Supplementary Figure 1. Gating strategies used for Flow Cytometry analysis. a. Gating strategy for T cells presented on Fig. 1b, 6a and supplementary 7. **b.** Gating strategy for CAR-T cells presented on Fig. 3e, 4a, and 6a. **c.** Gating strategy for CAR exosomes presented on Fig. 3e and 4a.

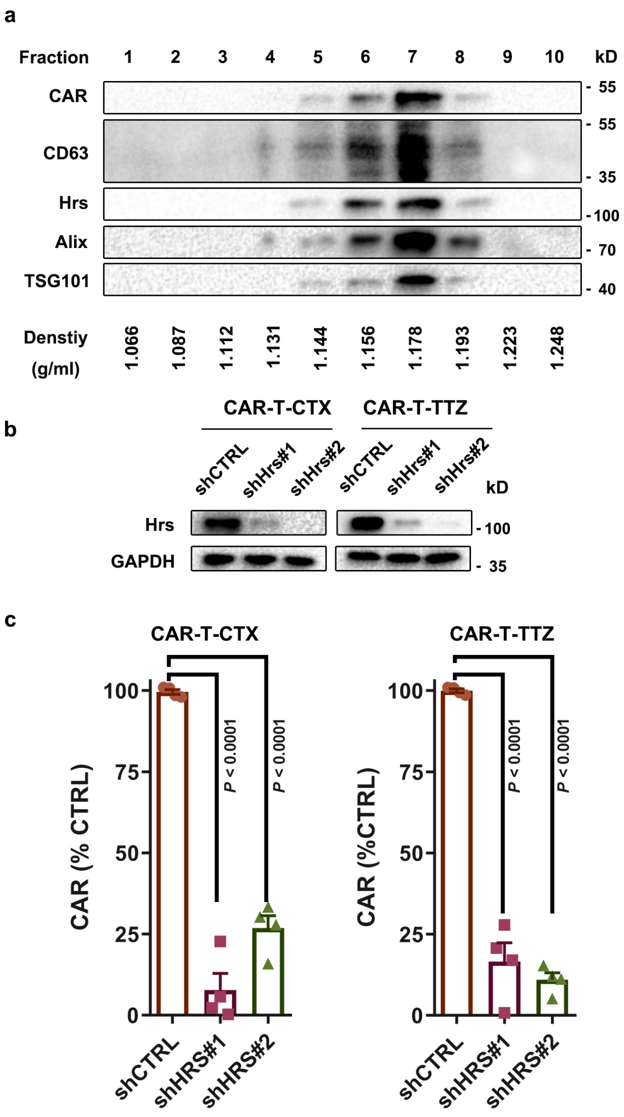

Supplementary Figure 2. CAR-T cells release extracellular vesicles carrying CAR. a. Density gradient centrifugation confirming that CAR secreted by CAR-T cells co-fractionated with exosome markers CD63, Hrs, Alix and TSG101. **b.** Western blot analysis of Hrs knockdown effect in different CAR-T cells. **c.** ELISA of CAR on exosomes from Hrs knockdown CAR-T cells with antigen expressing cell stimulation strategies (MDA-MB-231 cells or SK-BR-3 cells). Results shown represent three (**a, b**) independent experiments. Data are the means ± s.d. of four (**c**) independent biological replicates. P values are from a two-sided unpaired t-test (**c**). Source data (**a-c**) are provided as a Source Data file.

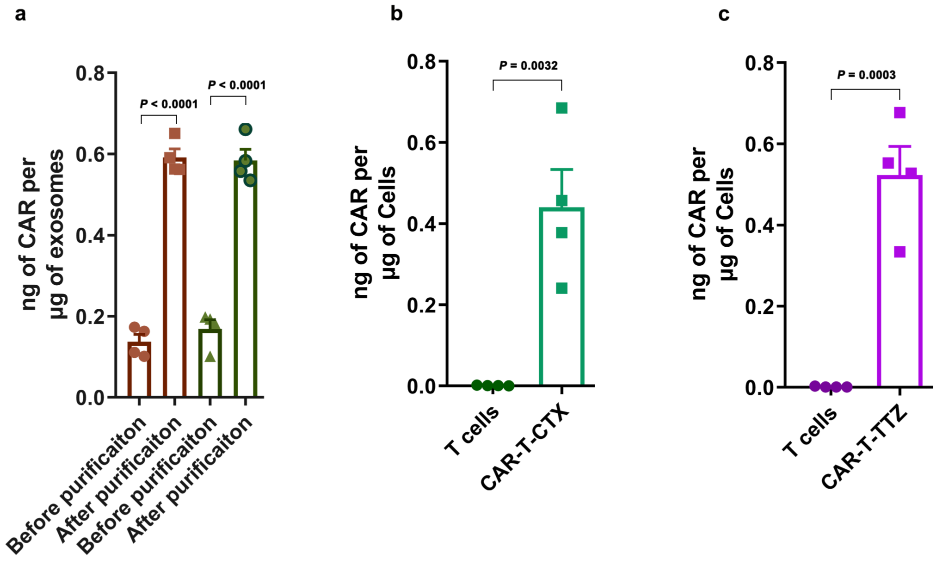

Supplementary Figure 3. CAR protein expression. a. ELISA of CAR on exosomes before and after purification. **b.** and **c.** ELISA of CAR on CAR-T Cells. Data are the means ± s.d. of four (**a-c**) independent biological replicates. P values are from a two-sided unpaired t-test (**a-c**). Source data (**a-c**) are provided as a Source Data file.

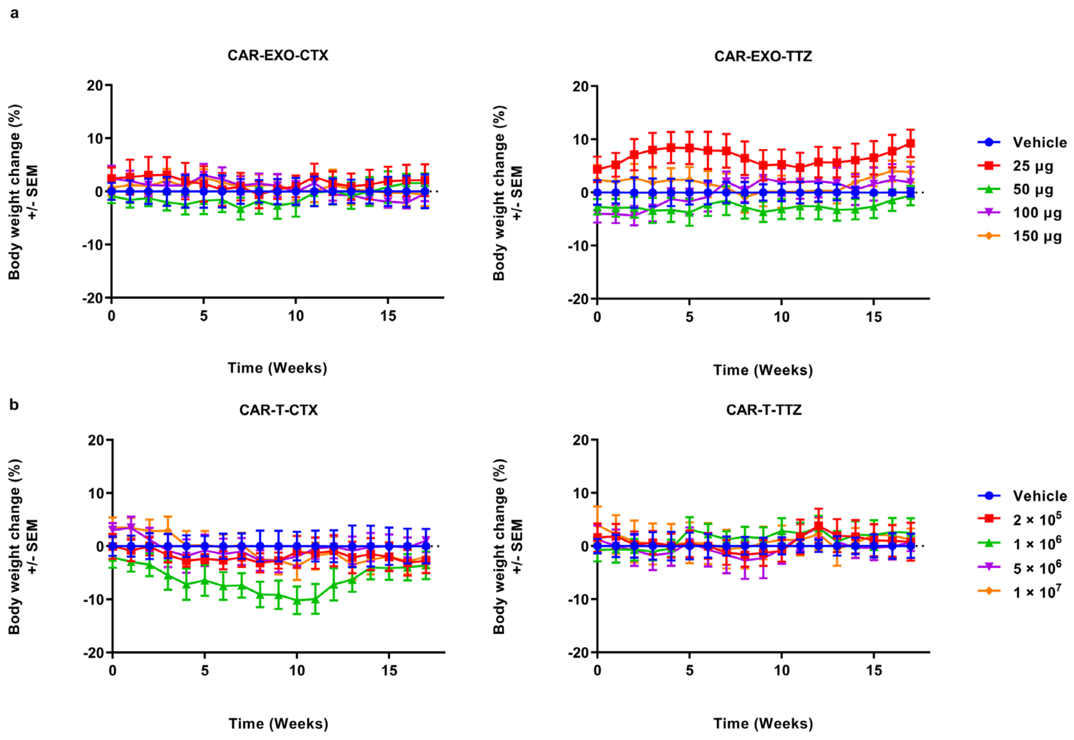

Supplementary Figure 4. Body weight change over time in response to different treatment. SCID mice (n=6) were treated with vehicle or different treatment (**a.**, Exosomes or **b.**, CAR-T cells), and the percentage of body weight change was measured weekly. Data are the means ± s.d. (**a-b**). Source data (**a-b**) are provided as a Source Data file.

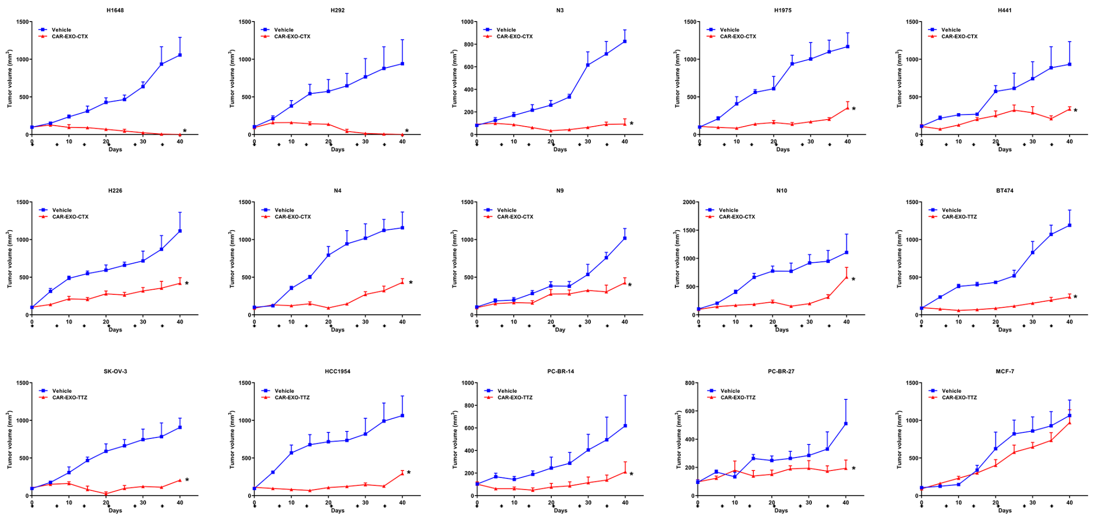

Supplementary Figure 5. CAR exosomes inhibited tumour growth. Cancer cell lines or patient-derived tumour tissue fragments established as subcutaneous xenografts (n = 8/group) and treated with weekly doses of CAR exosomes (100 µg). Data are means ± s.e.m. *P < 0.05 versus Vehicle by the nonparametric *t* test. Source data are provided as a Source Data file.

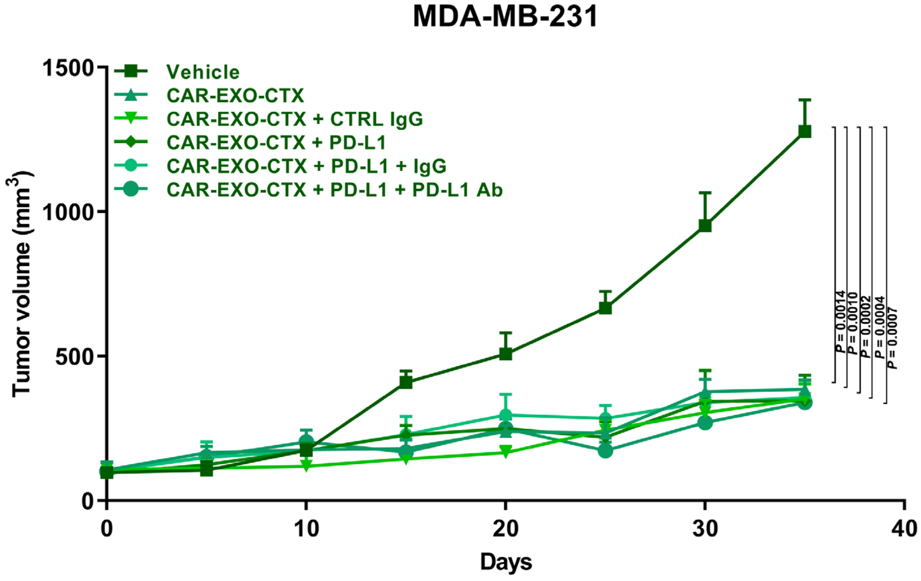

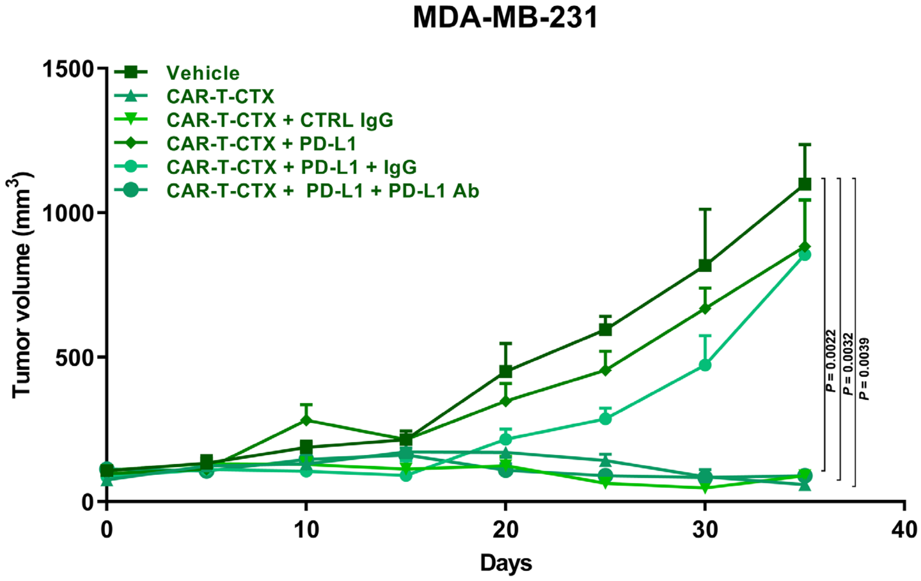

Supplementary Figure 6. PD-L1 inhibits CAR-T cells but not CAR exosomes in vivo. Tumour volumes of MDA-MB-231 tumour xenografts after the indicated treatment with intratumoural (i.t.) routes (up, CAR-exosomes; down, CAR-T cells). The data are represented as the means ± s.e.m.. Statistical analyses were performed by two-way ANOVA followed by Bonferroni post-test comparison. Source data are provided as a Source Data file.

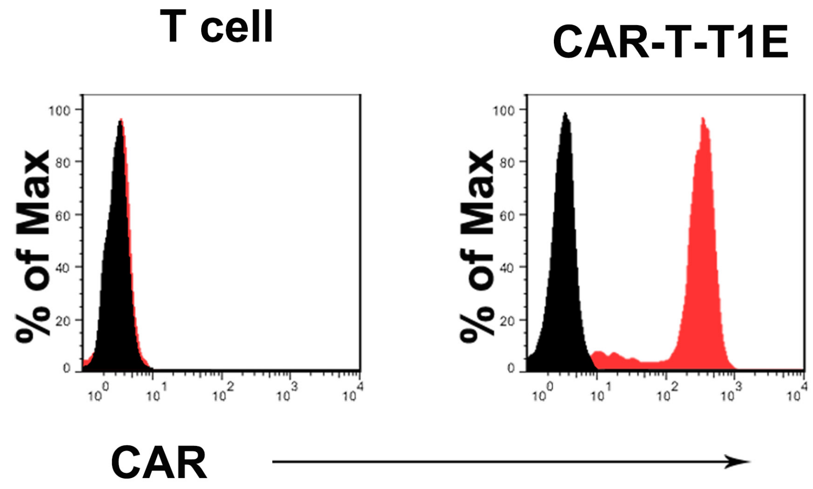

Supplementary Figure 7. CAR expression in CAR-T cells. Forty-eight hours after retroviral transduction, the expression of CAR on human T cells was detected by staining with anti-MYC antibody, followed by flow cytometry analysis. T cells without transduction were used as a negative control. The histograms shown in black correspond to the isotype controls, whereas the red histograms indicate the positive fluorescence. The experiment was repeated three times independently with similar results.

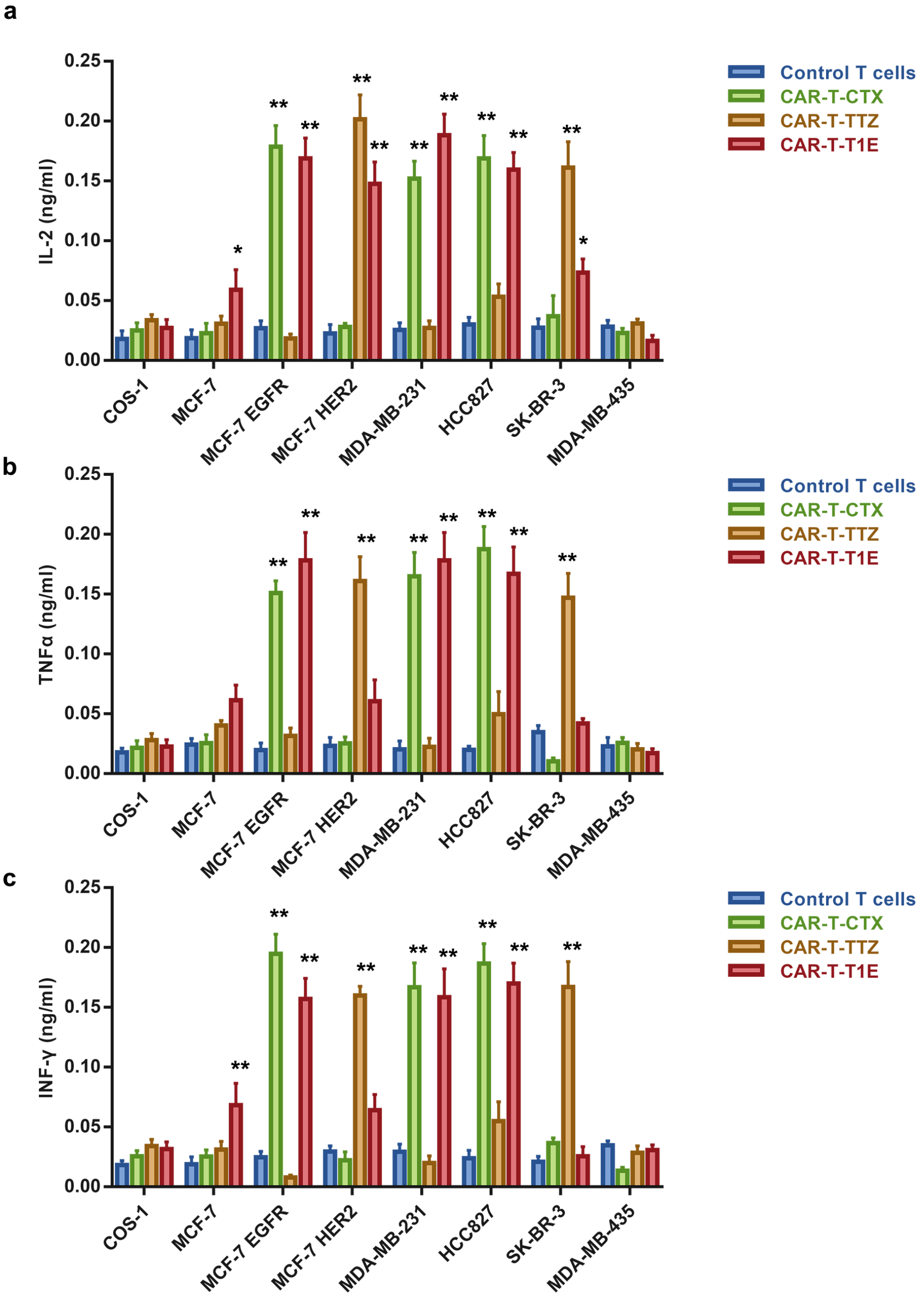

Supplementary Figure 8. Targeting specificity of different CAR-T cells. 1×10^6 of the indicated engineered T-cell populations was cocultivated with an equal number of indicated cancer cells that express the specified ErbB receptor(s). Supernatants were harvested at 48 h (a, IL-2 and b, TNFα) and 72 h (c, IFN-γ) for ELISA analysis. Data are presented as means ± s.d. of six independent biological replicates. *P < 0.05; **P < 0.01 versus control T cells by one-way ANOVA followed by Tukey post-test. Source data are provided as a Source Data file.

Supplementary Table 1. EGFR and HER2 receptor expression on cell lines[a]

Cancer cell line	Isotype control	EGFR	HER2
MCF-7	2.6	16.6	22.3
MCF-7 EGFR	2.4	395.5	27.5
MCF-7 HER2	2.5	24.6	358.5
MDA-MB-231	2.6	556.7	32.5
HCC827	2.5	451.6	196.5
SK-BR-3	2.9	155.4	702.3

a Receptor expression levels were determined by indirect immunofluorescence assays. Briefly, cells were incubated with anti-human EGFR antibody, anti-human ErbB2 antibody, or control mouse IgG for 1 h and then washed, followed by the addition of secondary antibody. After 1 h, the cells were washed and analyzed by flow cytometry.

Supplementary Table 2. Information of used antibodies

Antibody	Provider	Application	dilution	Identifier
Anti-Myc-tag	Cell Signaling Technology	FCM	1:500	#2276
CD63	Abcam	WB	1:500	#ab68418
Hrs	Cell Signaling Technology	WB	1:1000	#15087
Alix	Cell Signaling Technology	WB	1:1000	#2171
TSG101	Abcam	WB	1:1000	ab125011
GAPDH	Cell Signaling Technology	WB	1:1000	#5174
Prohibitin	Invitrogen	WB	1:200	#II-14-10
Calregulin	invitrogen	WB	1:500	#PA3-900
Golgi 58K	Abcam	WB	1:1000	#ab27043
nucleoporin p62	BD Biosciences	WB	1:2000	#610497
HLA A, B, C	BioLegend	FCM	1:20	#311404
CD3	Invitrogen	FCM	1:200	#16-0037-81
CXCR4	R&D Systems	FCM	1:200	#MAB170
CD57	Invitrogen	FCM	1:20	#MA5-12008
CD27	Invitrogen	FCM	1:100	#14-0271-82
CD28	BioLegend	FCM	1:100	#102102
CD45RA	BD Biosciences	FCM	1:5 (1 test)	#555488
PD1	Abcam	FCM	1:100	#ab52587
EGFR	Cell Signaling Technology	IF	1:50	#4267
HER2	Cell Signaling Technology	IF	1:100	#2165
Ki-67	Abcam	FCM	1:100	#ab15580
Granzyme B	BioLegend	FCM	1:20	#515405
Perforin	Santa Cruz	WB	1:500	#sc-373943
Granzyme B	Santa Cruz	WB	1:200	#sc-8022

WB: Western Blotting; FCM: Flow cytometry; IF: Immunofluorescence;

Supplementary Table 3. Primer sequences for real-time quantitative PCR

Gene	Forward	Reverse
IL-2	5’-CCTTGCACTTCTGAAGAGATTGA-3’	5’-ACAGGGCCATCATAAAAGAGGT-3’
IFN-γ	5’-TCGGTAACTGACTTGAATGTCCA-3’	5’-TCGCTTCCCTGTTTTAGCTGC-3’
TNF-α	5’-GAGGCCAAGCCCTGGTATG-3’	5’-CGGGCCGATTGATCTCAGC-3’
GAPDH	5’-CAACGGATTTGGTCGTATTG-3’	5’-GCAACAATATCCACTTTACCAGAGTTAA-3’
