# Supplementary material for: CAR exosomes derived from effector CAR-T cells have potent antitumour effects and low toxicity
Source: Nat Commun. 2019 Sep 25;10:4355. doi: 10.1038/s41467-019-12321-3 (PMC6761190; doi:10.1038/s41467-019-12321-3)
Supplement: Supplementary file 2 — Reporting Summary [file 41467_2019_12321_MOESM2_ESM.pdf]

nature research

Corresponding author(s): Shi Hu

Last updated by author(s): Aug 10, 2019

Reporting Summary

Nature Research wishes to improve the reproducibility of the work that we publish. This form provides structure for consistency and transparency in reporting. For further information on Nature Research policies, see Authors & Referees and the Editorial Policy Checklist.

Statistics

For all statistical analyses, confirm that the following items are present in the figure legend, table legend, main text, or Methods section.

n/a	Confirmed	
☐	☒	The exact sample size (*n*) for each experimental group/condition, given as a discrete number and unit of measurement
☐	☒	A statement on whether measurements were taken from distinct samples or whether the same sample was measured repeatedly
☐	☒	The statistical test(s) used AND whether they are one- or two-sided<br>*Only common tests should be described solely by name; describe more complex techniques in the Methods section.*
☒	☐	A description of all covariates tested
☐	☒	A description of any assumptions or corrections, such as tests of normality and adjustment for multiple comparisons
☐	☒	A full description of the statistical parameters including central tendency (e.g. means) or other basic estimates (e.g. regression coefficient) AND variation (e.g. standard deviation) or associated estimates of uncertainty (e.g. confidence intervals)
☐	☒	For null hypothesis testing, the test statistic (e.g. *F*, *t*, *r*) with confidence intervals, effect sizes, degrees of freedom and *P* value noted<br>*Give P values as exact values whenever suitable.*
☒	☐	For Bayesian analysis, information on the choice of priors and Markov chain Monte Carlo settings
☒	☐	For hierarchical and complex designs, identification of the appropriate level for tests and full reporting of outcomes
☒	☐	Estimates of effect sizes (e.g. Cohen's *d*, Pearson's *r*), indicating how they were calculated

Our web collection on statistics for biologists contains articles on many of the points above.

Software and code

Policy information about availability of computer code

Data collection	no custom software was used
Data analysis	no custom software was used

For manuscripts utilizing custom algorithms or software that are central to the research but not yet described in published literature, software must be made available to editors/reviewers. We strongly encourage code deposition in a community repository (e.g. GitHub). See the Nature Research guidelines for submitting code & software for further information.

Data

Policy information about availability of data

All manuscripts must include a data availability statement. This statement should provide the following information, where applicable:
- Accession codes, unique identifiers, or web links for publicly available datasets
- A list of figures that have associated raw data
- A description of any restrictions on data availability

The authors declare that the data supporting the findings of this study are available within the paper and its supplementary information files. If needed, additional information is available from the corresponding author upon reasonable request. no restrictions on data availability

Field-specific reporting

Please select the one below that is the best fit for your research. If you are not sure, read the appropriate sections before making your selection.

☒ Life sciences ☐ Behavioural & social sciences ☐ Ecological, evolutionary & environmental sciences

Life sciences study design

All studies must disclose on these points even when the disclosure is negative.

Sample size	The chosen sample size are based on the numbers used for previous publications, which is most optimal to generate statistically significant results.
Data exclusions	No data were excluded from the analyses.
Replication	All replicates reported in the manuscript are biological replicates. All the statistics reported in the manuscript are based on at least 3 biologically independent replicates. All attempts to replicate the experiments were successful.
Randomization	The samples/cells were randomized to be examined. For in vivo mouse xenograft experiments, the mice were randomly grouped prior to the treatments.
Blinding	For in vitro and in vivo studies, the experiments were performed in a blinded fashion when possible, which means that people performing the assays were not aware of the treatment groups until the data analyses were completed.

Reporting for specific materials, systems and methods

We require information from authors about some types of materials, experimental systems and methods used in many studies. Here, indicate whether each material, system or method listed is relevant to your study. If you are not sure if a list item applies to your research, read the appropriate section before selecting a response.

Materials & experimental systems

n/a	Involved in the study	
☐	☒	Antibodies
☐	☒	Eukaryotic cell lines
☒	☐	Palaeontology
☐	☒	Animals and other organisms
☐	☒	Human research participants
☒	☐	Clinical data

Methods

n/a	Involved in the study	
☒	☐	ChIP-seq
☐	☒	Flow cytometry
☒	☐	MRI-based neuroimaging

Antibodies

Antibodies used	Myc-tag (CST, #2276); CD63 (Abcam,#ab68418); Hrs (CST, #15087); Alix (CST, #2171); TSG101 (Abcam, #ab125011), GAPDH (CST, #5174); Prohibitin (Invitrogen, #II-14-10); Calregulin (invitrogen, #PA3-900); Golgi 58K (Abcam, #ab27043); nucleoporin p62 (BD Biosciences, #610497); HLA A, B, C (BioLegend, #311404); CD3 (Invitrogen, #16-0037-81); CXCR4 (R&D Systems; # MAB170); CD57 (Invitrogen, #MA5-12008); CD27 (Invitrogen; #14-0271-82); CD28 (BioLegend, #102102); CD45RA (BD Biosciences, Clone HI100); PD1 (Abcam, #ab52587); EGFR (CST, #4267); HER2 (CST, #2165); Perforin (Santa Cruz, #373943), Granzyme B (Santa Cruz, #8022)
Validation	Antibody validation was deferred to the manufacturers and was supported by multiple publications.

Eukaryotic cell lines

Policy information about cell lines

Cell line source(s)	All cell lines were purchased from the American Type Culture Collection (ATCC, Manassas, VA)
Authentication	The identities of the cell lines were verified by STR analysis if cell line is purchased 6 months ago.
Mycoplasma contamination	All the cell lines presented in this study were tested for mycoplasma contamination and they were free of mycoplasma contamination.
Commonly misidentified lines (See ICLAC register)	No commonly misidentified cell lines were used.

Animals and other organisms

Policy information about studies involving animals; ARRIVE guidelines recommended for reporting animal research

Laboratory animals	All Mice were obtained from the Animal Centre of Chinese Academy of Sciences

Wild animals	The study did not involve wild animals.
Field-collected samples	The study did not involve samples collected from the field.
Ethics oversight	All animals were treated in accordance with guidelines of the Committee on Animals of the Chinese Academy of Sciences.

Note that full information on the approval of the study protocol must also be provided in the manuscript.

Human research participants

Policy information about studies involving human research participants

Population characteristics	N/A
Recruitment	N/A
Ethics oversight	Primary human T cells from healthy volunteer donors were obtained from the Changhai Hospital of Second Military Medical University. The clinical protocol was approved by the Second Military Medical University Review Board, with informed consent was obtained from each donor. Patient specimens were obtained during initial surgery from primary-diagnosed, early-stage cancer patients at Changhai Hospital of Second Military Medical University. Written informed consent was obtained from each patient, and the local ethics committee of Changhai Hospital, Second Military Medical University approved experimental design and tissue samples collection.

Note that full information on the approval of the study protocol must also be provided in the manuscript.

Flow Cytometry

Plots

Confirm that:

☒ The axis labels state the marker and fluorochrome used (e.g. CD4-FITC).

☒ The axis scales are clearly visible. Include numbers along axes only for bottom left plot of group (a 'group' is an analysis of identical markers).

☒ All plots are contour plots with outliers or pseudocolor plots.

☒ A numerical value for number of cells or percentage (with statistics) is provided.

Methodology

Sample preparation	Purified exosomes were incubated with 4-µm-diameter aldehyde/sulphate latex beads (Interfacial Dynamics) in PBS overnight at 4°C under gentle agitation. Exosome or cell surface staining was performed for 30 min at 4 °C and was analysed.Intra-exosome or -cellular staining was performed for 60 min on ice after using a fixation/permeabilization kit (eBioscience).
Instrument	BD Bioscience LSR II cytometer
Software	FACSDiva software, CellQuest Software,FlowJo software
Cell population abundance	No sorting was performed
Gating strategy	Preliminary FSC/SSD gates were always performed and the negative/positive boundaries were obtained using negative controls such as isotype controls

☒ Tick this box to confirm that a figure exemplifying the gating strategy is provided in the Supplementary Information.
